# Association of Prenatal Acetaminophen Exposure With Risk of ADHD and ASD in Offspring: A systematic review and meta-analysis

**DOI:** 10.1192/j.eurpsy.2025.370

**Published:** 2025-08-26

**Authors:** D. Carrazzoni Godoi, K. Guedes Amorim, R. Góes de Oliveira Galvão, A. C. Putini Vieira, L. Bussiki Corrêa da Costa Kotecki, D. Abraham Batista da Hora, S. A. de Souza Júnior

**Affiliations:** 1 School of Psychology, Cardiff University, Cardiff, United Kingdom; 2 Medical Sciences Center, Federal University of Paraíba, João Pessoa; 3 Escola Superior de Ciências da Saúde, Brasilia; 4 Universidade Santo Amaro, Santo Amaro; 5 Centro Universitário de Várzea Grande, Várzea Grande; 6 Universidade Federal do Amazonas, Manaus; 7 Centro de Ciências da Saúde, Curso de Medicina, Universidade de Fortaleza; 8 Departament of Physiology and Pharmacology, Faculty of Medicine, Federal University of Ceara, Fortaleza, Brazil

## Abstract

**Introduction:**

The association between prenatal acetaminophen exposure and the development of Attention Deficit Hyperactivity Disorder (ADHD) and Autism Spectrum Disorder (ASD) remains a subject of considerable debate. Despite extensive research, the evidence regarding this relationship is conflicting.

**Objectives:**

To perform a systematic review and meta-analysis of studies comparing the incidence of ADHD and ASD in patients that were either exposed or not exposed to acetaminophen prenatally.

**Methods:**

We systematically searched Pubmed, Embase and Cochrane Central for eligible studies up until August 2024. Only studies which included participants with a medical diagnosis of ADHD/ASD and reported acetaminophen exposure as a binary measure were included. Available summary data was extracted from published reports and pooled with a random-effects model using odds ratios (OR) with 95% confidence intervals (CI). Hazard ratios (HR) adjusted for potential confounding factors were used for sensitivity analyses. All statistical analyses were conducted utilizing Review Manager 5.4.1. PROSPERO iD:CRD42024587662.

**Results:**

We included five studies with a total of 2,647,536 patients with ADHD (150,741) / ASD (63,726), of whom 271,126 were exposed to acetaminophen prenatally and 2,376,410 were not exposed. Prenatal acetaminophen exposure was associated with an increased risk of developing ADHD (OR 1.30; 95% CI 1.17 to 1.45; p<0.01; I2 = 73%; Figure 1) and ASD (OR 1.17; 95% CI 1.14 - 1.20; p<0.01; I2 = 0%; Figure 2). Sensitivity analyses revealed that acetaminophen exposure during the third trimester of pregnancy was associated with an increased risk of ADHD (HR 1.26; 95% CI 1.07 to 1.47; p<0.01; I2 = 0%; Figure 3), but not during first (HR 1.10; 95% CI 0.97 to 1.26; p=0.13; I2 = 0%; Figure 3) and second (HR 1.07; 95% CI 0.95 to 1.19; p=0.26; I2 = 0%; Figure 3) trimesters.

**Image 1:**

**Figure:**
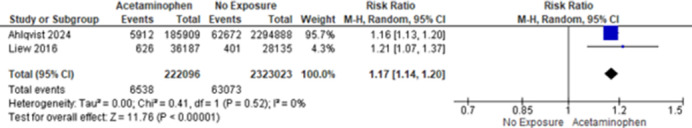

**Image 2:**

**Figure:**
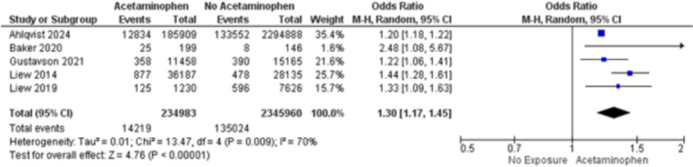

**Image 3:**

**Figure:**
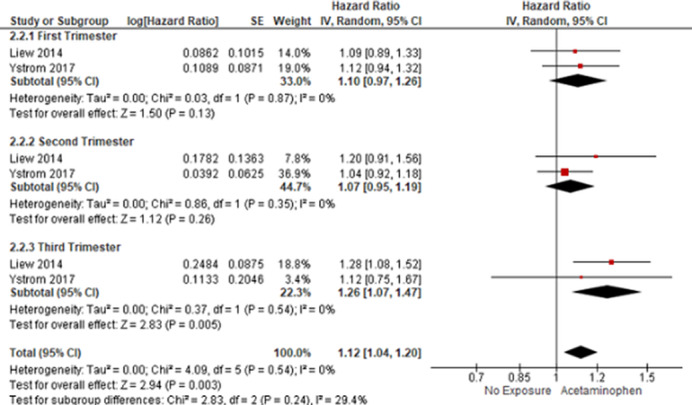

**Conclusions:**

In this systematic review and meta-analysis, prenatal acetaminophen exposure was significantly associated with risk of developing ADHD and ASD, especially if exposure occurs in the third trimester of pregnancy.

**Disclosure of Interest:**

None Declared

